# Clinical Efficacy of One Short Course of Mannan-Conjugated Birch Pollen Allergoid Immunotherapy: A Comparative Evaluation After Prior Placebo Treatment

**DOI:** 10.3390/jcm14238565

**Published:** 2025-12-03

**Authors:** Sandra del Pozo, Natascha Wahrhusen, Oliver Pfaar, Esther Raskopf, Anna Rybachuk, Cengizhan Acikel, Hacer Sahin, Silke Allekotte, José Luis Subiza, Miguel Casanovas, Mandy Cuevas, Ralph Mösges

**Affiliations:** 1 Inmunotek S.L., Calle Punto Mobi 5, Alcalá de Henares, 28805 Madrid, Spain; sdelpozo@inmunotek.com (S.d.P.);; 2 ClinCompetence Cologne GmbH, 50668 Cologne, Germany; 3 Institute of Medical Statistics and Computational Biology, Faculty of Medicine, University of Cologne, Kerpener Str. 62, 50937 Cologne, Germany; 4 Department of Otorhinolaryngology, Head and Neck Surgery, Section of Rhinology and Allergy, University Hospital Marburg, Philipps-Universität Marburg, 35037 Marburg, Germany; 5 Department of Otorhinolaryngology, Head and Neck Surgery, Faculty of Medicine (and University Hospital) Carl Gustav Carus, TUD Dresden University of Technology, 01069 Dresden, Germany

**Keywords:** birch pollen allergy, AIT, mannan-conjugated birch pollen allergoid, SCIT, combined symptom and medication score, CSMS

## Abstract

**Background/Objectives**: Allergen immunotherapy (AIT) with chemically modified allergoids represents a promising approach for the treatment of allergic rhinoconjunctivitis. This post hoc analysis investigated the clinical efficacy of a mannan-conjugated birch pollen allergoid (T502) by comparing outcomes in patients who received placebo in one pollen season and active treatment in the following year. **Methods**: Data were derived from four randomized clinical trials (EudraCT Numbers: 2018-002522-23, 2020-004126-32, 2021-002252-36, and 2022-004082-20) conducted over four consecutive years. Two independent patient groups were analyzed, with each group receiving placebo in the first year and subcutaneous T502 (10,000 mTU/mL) in the subsequent year. Daily symptom score (dSS), daily medication score (dMS), and combined symptom and medication score (CSMS) were assessed during the birch pollen season in April of each year. **Results**: In the first comparison between placebo in 2020 and an early treatment start in January 2021, treatment with T502 led to a 43.79% reduction in dSS (*p* ≤ 0.0001), a 71.43% reduction in dMS (*p* ≤ 0.001), and a 42.1% reduction in CSMS (*p* ≤ 0.001), all statistically significant. In the second comparison between placebo in 2022 and a late treatment start in March 2023, the reduction in dMS was 74.47% (*p* ≤ 0.001), whereas reductions in dSS (1.47%, *p* = 0.898) and CSMS (20.3%, *p* = 0.057) were not statistically significant. **Conclusions**: Subcutaneous immunotherapy (SCIT) with T502 significantly reduces allergic symptoms and medication use, particularly when all injections are completed before the onset of the birch pollen season in April. These findings highlight both the clinical value of T502 and the importance of optimized treatment timing.

## 1. Introduction

Birch pollen (Betula) allergy is the most widespread tree pollen allergy in regions such as Northern and Central Europe [1,2], as well as China [3]. Sensitization to birch pollen affects approximately 8% to 16% of the European population, with regional differences in prevalence [2]. Recent studies indicate a rising prevalence of allergic rhinitis, especially in regions where it was previously less widespread [4]. The development of allergic rhinitis is likely the result of multiple factors, including genetic predisposition [5,6].

Allergic rhinitis (AR) is primarily triggered by an immunoglobulin E (IgE)-mediated hypersensitivity reaction following allergen exposure [7]. The condition was first defined in 1929 by three cardinal symptoms: sneezing, nasal congestion, and mucus secretion [8]. Beyond these physical symptoms, AR can significantly affect social life, academic performance, and work productivity [9,10]. 

Effective management is therefore essential—not only to alleviate symptoms but also to address associated or complicating respiratory conditions such as asthma, sinusitis, and sleep apnea [11]. In addition to pollen avoidance, which is not always possible, anti-allergic medications are commonly used to alleviate allergy symptoms [12]. However, they require continuous use for as long as symptoms persist, which is often lifelong [13]. Unlike symptomatic therapies, AIT remains the only disease-modifying approach for allergic respiratory conditions [14,15,16].

One challenge of AIT is poor patient adherence [17], as the treatment requires numerous injections over 3–5 months and continuation of administration for at least three years is advised [18]. A study by Scadding et al. (2017) examined a two-year course of either subcutaneous or sublingual grass pollen immunotherapy but found no sustained long-term benefit [19]. Supporting the benefit of a treatment duration of at least three years, a study by Durham et al. showed that three to four years of grass pollen immunotherapy led to long-term clinical remission and sustained immunologic changes, with symptom and medication scores remaining low even after discontinuation [20].

Side effects can occur with the initial doses, often leading to early discontinuation [21]. These limitations highlight the urgent need for safer and more effective AIT to improve patient compliance and therapeutic outcomes [22].

To optimize the efficacy of AIT and shorten treatment duration, allergens modified with glutaraldehyde or formaldehyde, known as allergoids, have been developed [23]. This modification improves immunogenicity properties by reacting with the primary amino groups in the allergen’s polypeptide chain to form high-molecular-weight cross-linked polymers, inactivating the conformational IgE epitopes while preserving the linear T-cell epitopes [21,24]. This structure allows the administration of high doses during a short accumulation phase [21,25]. The efficacy of allergoids has been further enhanced by combining them with adjuvants that people are exposed to in everyday life such as mannan, a polymannose backbone derived from yeast and a natural ingredient of bread and beer [25,26]. An adjuvant is a molecule that amplifies the immune response through physical or chemical interaction with antigens [21,27]. In this context, allergoids are conjugated to non-oxidized mannan—typically using glutaraldehyde as a cross-linker—which enables targeted delivery to dendritic cells via C-type lectin receptors. This targeted approach aims to increase the bioavailability of the administered dose [22].

T502 is a mannan-conjugated birch pollen allergoid consisting of a glutaraldehyde-polymerized birch pollen extract coupled to mannan from Saccharomyces cerevisiae. In the first human trial (T502-SIT-020), all tested doses were found to be safe; however, the highest dose of 10,000 mTU (mannan therapeutic units)/mL demonstrated the highest efficacy and was therefore selected for subsequent studies [28].

This study analyzes data from four clinical trials—two of which were follow-up studies—investigating the efficacy of AIT with T502. The focus is on patients who received placebo in one year and active therapy in the next. Symptom improvement and medication use are assessed. Notably, one follow-up study administered AIT partly during the birch pollen season, allowing comparison with the earlier study with pre-seasonal treatments only. This allows us to draw conclusions on the optimal timing of therapy.

## 2. Materials and Methods

### 2.1. T502-SIT-020 Study (EudraCT Number: 2018-002522-23) [28]

Conducted in 2020, this phase II, randomized, double-blind, placebo-controlled dose-finding study evaluated the safety and efficacy of pre-seasonal subcutaneous immunotherapy with mannan-conjugated birch pollen allergoids (T502) in patients with birch pollen-induced allergic rhinoconjunctivitis (ARC).

Patients received one of three T502 concentrations (1000 (n = 62)/3000 (n = 62)/10,000 (n = 61) mTU/mL) or placebo (n = 61). Symptoms and medication use during the pollen season were analyzed.

**Figure 1:**
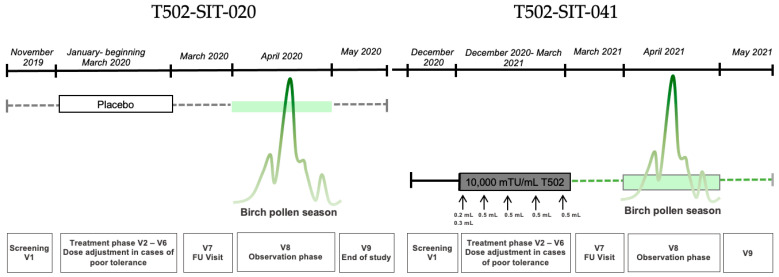
Study design for a patient receiving placebo in the T502-SIT-020 study in 2020, with nine visits, and receiving active treatment afterwards in the T502-SIT-041 study in 2021, also with nine visits.

The study included nine visits: one screening visit, five pre-seasonal treatment visits, one follow-up, and two visits during and after the peak pollen season (Figure 1). Subcutaneous injections were administered at intervals of 7 to 30 days between visits V2 and V6 prior to the pollen season. Each dose consisted of 0.5 mL, divided into two injections during the first visit: 0.2 mL in one arm and 0.3 mL in the other after a 30 min interval. In total, 246 patients were randomized into one placebo group and three active treatment groups with different T502 concentrations.

### 2.2. T502-SIT-041 Study (EudraCT Number: 2020-004126-32)

This open-label, phase II follow-up study, conducted in 2021–2024, included patients from the previous T502-SIT-020 study. For the study presented herein, only data from patients who received placebo in the previous study (2020) were analyzed. All participants received the recommended dose of 10,000 mTU/mL T502, regardless of their prior group assignment.

**Figure 2:**
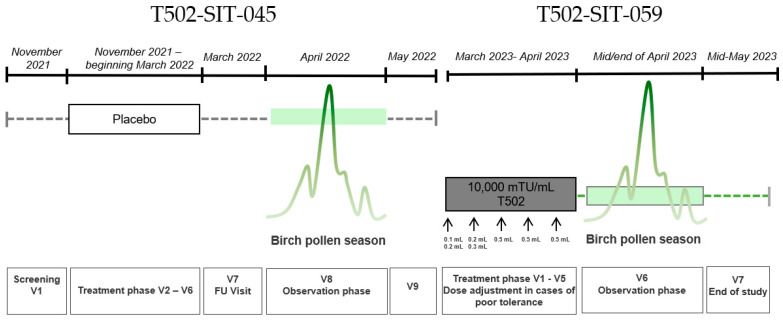
Study design for a patient receiving placebo in the T502-SIT-045 study in 2022, with nine visits, and receiving active treatment afterwards in the T502-SIT-059 study in 2023, with seven visits.

Nine visits were scheduled in the first year of the study: one screening visit, five pre-seasonal treatment visits, one follow-up, and two visits during and after the peak pollen season (Figure 2). During pre-seasonal treatment, patients received five subcutaneous injections of 10,000 mTU/mL T502 at two-week intervals (±6 days). On the first visit, the 0.5 mL dose was split into two injections; on subsequent visits, it was administered as a single injection. Between December 2020 and May 2021, a total of 154 patients from the T502-SIT-020 study were treated with T502 in this open-label trial.

### 2.3. T502-SIT-045-Study (EudraCT-Number: 2021-002252-36)

The T502-SIT-045 study (2022) was a prospective, randomized, double-blind, placebo-controlled, multicenter phase III trial evaluating the efficacy of subcutaneous T502 (10,000 mTU/mL) in patients with birch pollen-induced ARC. A total of 298 patients were randomized to receive either T502 (n = 199) or placebo (n = 99) prior to the birch pollen season. Treatment was administered as follows: 1st treatment visit: 0.1 + 0.2 mL; 2nd treatment visit: 0.2 + 0.3 mL; all other treatment visits: 0.5 mL across five pre-seasonal visits, with split dosing during the first two visits based on previous study protocols. For the study presented herein, only data from patients who received placebo were analyzed. Symptoms and medication use were assessed during the peak birch pollen season.

### 2.4. T502-SIT-059-Study (EudraCT-Number: 2022-004082-20)

The T502-SIT-059 study (2023) was an open-label, phase III, follow-up trial of patients who were previously enrolled in the T502-SIT-045 study. All participants received subcutaneous T502 (10,000 mTU/mL) to evaluate its continued clinical efficacy. A total of seven visits were conducted, with five treatment visits and two visits during and after the birch pollen season. Treatment was administered as follows: 1st treatment visit: 0.1 + 0.2 mL; 2nd treatment visit: 0.2 + 0.3 mL; all other treatment visits: 0.5 mL across five visits, with split dosing during the first two visits. Symptoms and medication use during the 2023 birch pollen season were compared with results from the 2022 season. The study aimed to provide all patients with active treatment and assess year-over-year improvements in allergic symptom control. In the T502-SIT-041 study (standard regimen), injections were planned every 2–4 weeks, with most patients receiving them approximately every 3 weeks. Since treatment was initiated in December, the total duration of the immunotherapy course was about 3–4 months. In contrast, in the T502-SIT-059 study (shorter regimen), injections were planned every 1–2 weeks and the majority of patients received them at 1-week intervals. As treatment began in March, the total duration was less than one month, and for some patients, the last injections were administered during the pollen season.

### 2.5. Endpoints

During the birch pollen season, patients in the T502-SIT-020 and T502-SIT-041 studies recorded their dSS and dMS using the mobile app CSMS+. For the T502-SIT-045 and T502-SIT-059 studies, the CSMS+ mobile app was also used.

In all four studies, the dSS was calculated based on six symptoms: four nasal (rhinorrhea, sneezing, nasal itching, and nasal congestion) and two ocular symptoms (ocular itching and watery eyes). Each symptom was rated daily using a 4-point ordinal scale:

0 = no symptoms;
1 = mild symptoms (clearly present but minimal perception; easy to tolerate);
2 = moderate symptoms (clearly bothersome but tolerable);
3 = severe symptoms (difficult to tolerate; interferes with daily activities and/or sleep).
The scores of the six symptoms were summed and divided by six to calculate the dSS, resulting in a score between 0 and 3.

The dMS reflected the highest level of medication used per day. Initially, for the T502-SIT-020 and T502-SIT-041 studies, a stepwise approach was applied, as follows:

0 = no medication;
1 = use of antihistamine tablets;
2 = use of nasal corticosteroids (with or without antihistamines);
3 = use of oral corticosteroids (with or without nasal corticosteroids and antihistamines).
The CSMS was calculated by adding the dSS and dMS as follows: CSMS = dSS (0–3) + dMS (0–3), resulting in a score in the range of 0 to 6 [29].

In the T502-SIT-045 and T502-SIT-059 studies, an expanded additive version of the dMS was introduced for improved differentiation, given that the use of oral corticosteroids was reported in fewer than 1% of patients in the previous studies [30]:

0 = no medication;
0.5 = use of antihistamine eye drops;
1 = use of antihistamine tablets;
1.5 = use of nasal corticosteroids.

### 2.6. Patient Groups

For this analysis, the patient identification numbers of all individuals who received placebo treatment in 2020 (T502-SIT-020) or 2022 (T502-SIT-045) and subsequently participated in the follow-up studies (T502-SIT-041 and T502-SIT-059, respectively) in the following year were compiled. The goal was to compare their CSMS, dSS, and dMS for the month of April over the years.

The dSS and dMS were recorded daily via an electronic patient diary and used to calculate the CSMS. This yielded 30 daily values per patient for each score during April. These values were averaged to obtain a monthly value for dSS, dMS, and CSMS for each patient. Each patient’s monthly value from the placebo year was then compared to their corresponding value in the following treatment year.

In total, 32 patients who received placebo in T502-SIT-020 and active treatment in T502-SIT-041 were identified, forming the T502-SIT-020–T502-SIT-041 comparison group. For the comparison, individual CSMS, dSS, and dMS values were analyzed. Similarly, 32 patients from the T502-SIT-045 placebo group who received AIT in T502-SIT-059 were included in the T502-SIT-045–T502-SIT-059 comparison group. For this comparison, the mean values for dSS and the additive dMS were used to calculate the CSMS, based on the additive scoring system introduced in 2022.

### 2.7. Statistical Analysis

Statistical analysis was performed using SPSS version 30 (Armonk, NY, USA). For the evaluation of continuous variables, the median, mean, minimum and maximum values, standard deviation, and quartiles were calculated. Two statistical tests were applied to assess significant difference, with the threshold for statistical significance set at α = 0.05.

In cases of normally distributed data, the mean was used as the central tendency measure, and statistical significance was determined using the *t*-test. For data that did not meet the assumption of normality, the median was used and significance was assessed using the Wilcoxon signed-rank test.

## 3. Results

### 3.1. Patients

A total of 64 patients were included in this post hoc analysis and data came from two groups of 32 patients each. These groups included a group of 32 patients who participated in the T502-SIT-020 and T502-SIT-041 studies in the years 2020 and 2021, respectively, and a second group of 32 patients who participated in the T502-SIT-045 and T502-SIT-059 studies in the years 2022 and 2023, respectively. The two study cohorts were very similar in their demographic composition (see Tables S5 and S6 in Supplementary Materials). The mean pollen exposure was high but similar across the four years of the studies (Figure S1).

### 3.2. Combined Symptom and Medication Score

**Figure 3:**
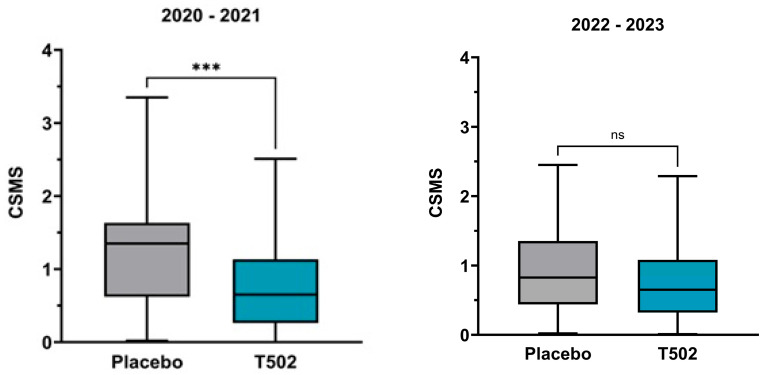
Changes in the median and interquartile CSMS. (**Left**)—patients who received placebo in the T502-SIT-020 study in 2020 and active treatment in the T502-SIT-041 study in 2021; (**right**)—patients who received placebo in the T502-SIT-045 study in 2022 and a shorter regimen treatment in the T502-SIT-059 study in 2023. *** *p* ≤ 0.001, ns indicates non-significant.

The CSMS (Figure 3) showed a clear treatment effect in the comparison between T502-SIT-020 and T502-SIT-041, with the mean CSMS decreasing significantly by 42.1%, from 1.31 to 0.784 (*t*-test *p* ≤ 0.001).

In the second comparison between T502-SIT-045 and T502-SIT-059, the mean CSMS decreased by 20.3%, from 0.95 to 0.7572 (Table S1), but this change did not reach statistical significance (*t*-test *p* = 0.057).

### 3.3. Daily Symptom Score

In the comparison between the T502-SIT-020 and T502-SIT-041 studies, a significant reduction in dSS was observed in patients who received T502 in the year following placebo treatment, with the median decreasing by 43.79% (Table S2) (*p* ≤ 0.0001).

**Figure 4:**
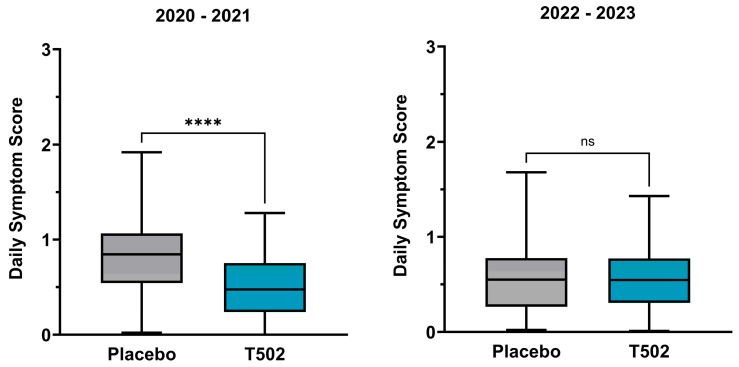
Changes in the median and interquartile dSS. (**Left**)—patients who received placebo in the T502-SIT-020 study in 2020 and active treatment in the T502-SIT-041 study in 2021; (**right**)—patients who received placebo in the T502-SIT-045 study in 2022 and a shorter regimen treatment in the T502-SIT-059 study in 2023. **** *p* ≤ 0.0001, ns indicates non-significant.

In contrast, the comparison between the T502-SIT-045 and T502-SIT-059 studies showed no meaningful change, with a non-significant reduction of 1.47% in the mean dSS (*t*-test *p* = 0.898) (Figure 4).

### 3.4. Daily Medication Score

**Figure 5:**
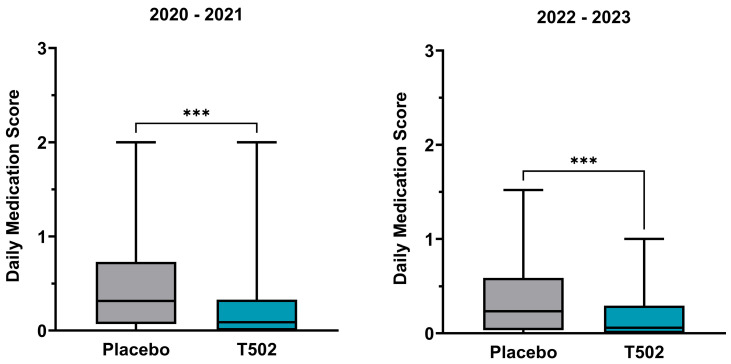
Changes in the median and interquartile dMS. (**Left**)—patients who received placebo in the T502-SIT-020 study in 2020 and active treatment in the T502-SIT-041 study in 2021; (**right**)—patients who received placebo in the T502-SIT-045 study in 2022 and a shorter regimen treatment in the T502-SIT-059 study in 2023. *** *p* ≤ 0.001.

The dMS showed a statistically significant reduction following treatment with T502 in both study comparisons (Table S3). In the comparison between T502-SIT-020 and T502-SIT-041, the median dMS was reduced by 71.43%, from 0.315 to 0.09 (Wilcoxon *p* ≤ 0.001) (Figure 5). In the second comparison between T502-SIT-045 and T502-SIT-059, treatment with T502 led to a 74.47% decrease in the median dMS, from 0.235 to 0.06 (Wilcoxon *p* ≤ 0.001) (Table S4).

## 4. Discussion

According to the World Allergy Organization (WAO), any additional efficacy lower than that achieved with antihistamines is considered insufficient. Therefore, a minimum clinically relevant effect requires the demonstration of at least a 20% improvement over placebo [31].

By taking into account the reductions in both dSS and dMS, the CSMS provides a comprehensive measure of treatment efficacy.

The results of this study indicate short-term clinical efficacy for T502, as assessments were performed during the first pollen season following treatment completion. However, because two regimens—the standard regimen and a shorter regimen—were evaluated, distinct immunological mechanisms acting at different temporal scales may be at play [32,33]. The standard regimen (T502-SIT-041) was administered over a 3–4-month pre-seasonal period, allowing adaptive mechanisms, such as the induction of allergen-specific IgG4, attenuation of Th2 responses, and expansion of regulatory T and B cells, to develop before allergen exposure. In contrast, the shorter regimen (T502-SIT-059) consisted of weekly administrations over a period of less than a month, favoring mainly rapid desensitization rather than full adaptive modulation. These temporal differences may account for the greater clinical benefit observed with the standard regimen. For the shorter regimen, adaptive responses may appear later and become evident in subsequent seasons, but this was not captured in the present analysis, which focused on short-term outcomes. These findings are consistent with the results reported by Zielen et al. (2025) [34], who found that an extended pre-seasonal regimen with wider dosing intervals showed superior clinical efficacy compared with shorter schedules, supporting the benefit of longer treatment duration before allergen exposure.

In a study by Pfaar et al. (2019), the primary endpoints after 3 to 6 months of sublingual immunotherapy (SLIT) treatment showed a significant and clinically relevant reduction of 32% in CSMS compared to placebo [16]. Similar results were reported in a 2013 study by Pfaar et al. on SCIT with a mixed depigmented-polymerized birch and grass pollen extract, where a 33.7% lower CSMS was observed in the second treatment year compared to placebo [35].

The dSS showed a statistically significant median reduction of 43.79% in the first comparison (T502-SIT-020 and T502-SIT-041). Gallego et al. also reported that AIT using depigmented, glutaraldehyde-modified allergens is highly effective in allergy treatment, demonstrating a 54% reduction in symptom scores compared to placebo [36]. A similarly high effect was observed in the present study, though the observed mean improvement of 1.47% in the second comparison was minimal and not statistically significant. One possible explanation is the timing of treatment, as the shorter regimen in the T502-SIT-059 study was partly administered during April, which might have prevented the AIT from exerting its full effect in time before the start of the birch pollen season.

In contrast to the variability observed in symptom scores, the dMS showed a consistent and substantial reduction in both comparisons. A median decrease of 71.43% was observed in the first group, and an even greater median reduction of 74.47% in the second. These findings underline the therapeutic benefit of T502, particularly in reducing the need for further medication across different treatment conditions.

As a comparison, Biedermann et al. (2019) found a similar effect on SLIT for ARC caused by tree pollen: differences of 37% in the dSS and 49% in the dMS were reported between the treatment and placebo groups [12]. The symptom reduction observed in the comparison between T502-SIT-020 and T502-SIT-041 reached 43.79%, exceeding the effect observed by Biedermann et al.

### 4.1. Limitations

A methodological limitation is the use of different statistical measures: the median was calculated for one endpoint due to the non-normal data distribution, while for endpoints with normal data distribution, the mean value was calculated, as shown in the comparison of the dSS (Table S4).

The CSMS has inherent limitations that concern the two components used to calculate the score. The assessment of symptoms is influenced by individual differences in allergen exposure, variability in daily symptom burden, and the retrospective nature of symptom reporting [37]. Similarly, the evaluation of daily medication intake within the CSMS can be limited by insufficient differentiation of medication types and usage patterns. To address this, in the present study, the second comparison applied the previously described additive CSMS approach, allowing for greater granularity in scoring. However, this may impact direct comparability between the two study groups. The use of such additive models has been recommended by Pfaar et al. [38] for future trials. Nonetheless, further research is needed to explore and validate additional clinical parameters to establish the most appropriate and reliable outcome measures for assessing AIT efficacy in clinical settings [39].

Our findings pertain to short-term clinical efficacy; earlier initiation with the standard regimen likely enhances the contribution of adaptive mechanisms that developed prior to the first allergy season, and the relationship between these short-term effects and longer-term sustainability (classically achieved with multi-year AIT) remains to be established in longitudinal studies assessing immunologic correlates.

### 4.2. COVID-19

It is noteworthy that the use of face masks and the reduction in air pollution during COVID-19 lockdowns are considered potential factors in alleviating symptoms of AR [40,41]. Face masks not only reduce pathogen exposure but also limit inhalation of airborne allergens such as pollen (10–100 µm), which are key triggers of IgE-mediated allergic responses. Surgical masks can filter particles as small as 3 µm, while N95 masks provide even greater protection, retaining particles down to 0.04 µm [41]. A study by Mengi et al. [42] showed that face mask use significantly reduced nasal symptoms in pollen allergy sufferers, with a 36% decrease in moderate to severe complaints.

Since all four studies took place in April of different years, variations in mask usage or pandemic-related behavioral changes between years could have influenced the dMS, dSS, or CSMS outcomes and may represent relevant confounding factors. It has been reported that the pandemic had an impact on AR treatment [43,44].

### 4.3. Implications for Future Research

Several conclusions can be drawn from the results of this post hoc analysis, which may be of relevance for future research. The data suggest that symptom development is more sensitive to the timing of AIT, with delayed administration resulting in a less pronounced reduction in symptoms. In contrast, the reduction in medication use appears robust even when treatment begins later, with a minimal difference of only 3.05% observed between the comparison groups. This indicates that considerable clinical benefit—particularly in terms of reduced medication need—can still be achieved even with a delayed treatment start.

Nonetheless, the greatest improvement in CSMS appears to occur when there is sufficient time between the initiation of AIT and the onset of the pollen season. Further studies are required to determine the optimal timing of T502 administration. It should also be noted that external factors, such as behavioral changes during the COVID-19 pandemic, may have influenced the results and should be more thoroughly addressed in future investigations.

Overall, the results from both comparison groups demonstrate that treatment with T502 leads to improvements, reflected in a significant reduction in allergic symptoms in the first group and a reduced need for medication in both groups. This translates into meaningful improvements in quality of life for patients during the pollen season [45], which could be observed already after the first year of treatment, and highlights T502 as a valuable therapeutic option for pollen allergy.

## 5. Conclusions

This study demonstrates the therapeutic potential of T502 AIT in treating birch pollen-induced ARC. While symptom scores proved more sensitive to treatment timing, medication scores showed robust improvement regardless of the injection schedule, underscoring the clinical benefit of T502 even with a delayed treatment onset that begins shortly before the start of the birch pollen season.

## Data Availability

Data will be available from the corresponding author upon reasonable request.

## Supplementary Materials

The following supporting information can be downloaded at: https://www.mdpi.com/article/10.3390/jcm14238565/s1, Figure S1: Mean pollen exposure across the 4 years of the studies; Table S1: Descriptive statistics of the CSMS for the T502-SIT-020, T502-SIT-041, T502-SIT-045, and T502-SIT-059 studies, showing mean/median reduction for the comparison between T502-SIT-020 and T502-SIT-041 or between T502-SIT-045 and T502-SIT-059; Table S2: Descriptive statistics of the dSS for the T502-SIT-020, T502-SIT-041, T502-SIT-045, and T502-SIT-059 studies, showing mean/median reduction for the comparison between T502-SIT-020 and T502-SIT-041 or between T502-SIT-045 and T502-SIT-059; Table S3: Descriptive statistics of the dMS for the T502-SIT-020, T502-SIT-041, T502-SIT-045, and T502-SIT-059 studies, showing mean/median reduction for the comparison between T502-SIT-020 and T502-SIT-041 or between T502-SIT-045 and T502-SIT-059; Table S4: Significance tests, with t-test for normally distributed data and Wilcoxon test for non-normally distributed data; Table S5: Baseline characteristics; Table S6: Demographic data (gender distribution and ethnic origin).

## Abbreviations

AIT	Allergen-specific immunotherapy
AR	Allergic rhinitis
ARC	Allergic rhinoconjunctivitis
COVID-19	Coronavirus disease 2019
CSMS	Combined symptom and medication score
dMS	Daily medication score
dSS	Daily symptom score
IgE	Immunoglobulin E
mTU	Mannan therapeutic units
*p*	(*p*-value) value for significance
SCIT	Subcutaneous immunotherapy
SLIT	Sublingual immunotherapy
SPSS	Statistical Package for the Social Sciences
V	Visit
WAO	World Allergy Organization
