# Supplementary material for: Clinical Efficacy of One Short Course of Mannan-Conjugated Birch Pollen Allergoid Immunotherapy: A Comparative Evaluation After Prior Placebo Treatment
Source: J Clin Med. 2025 Dec 3;14(23):8565. doi: 10.3390/jcm14238565 (PMC12692785; doi:10.3390/jcm14238565)
Supplement: Supplementary file 1 [file jcm-14-08565-s001.zip › jcm-3974958-supplementary.pdf]

Supplementary Materials:

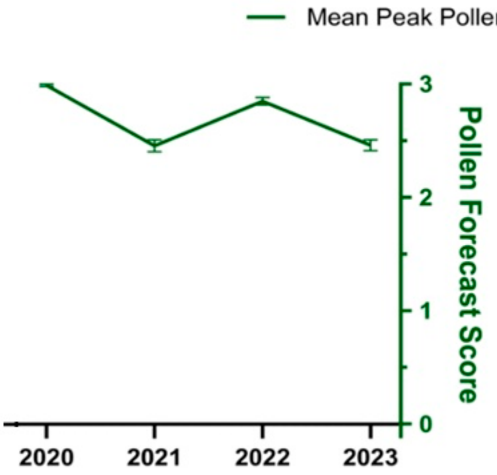

Figure S1. Mean pollen exposure across the 4 years of the studies.

Classification levels are as follows:

0 = No exposure: There is no or very little pollen in the air.

1 = Low exposure: The pollen concentration is low but noticeable.

2 = Medium exposure: There is a medium concentration of pollen.

3 = High exposure: The pollen concentration is high, which can lead to more severe symptoms for allergy sufferers.

Table S1. Descriptive statistics of the CSMS for T502-SIT-020, T502-SIT-041, T502-SIT-045, and T502-SIT-059 (shorter regimen), showing mean/median reduction for the comparison between T502-SIT-020 and T502-SIT-041 or between T502-SIT-045 and T502-SIT-059.

Study	N	Mean	Standard deviation	Median	25th Percentile	75th Percentile	Minimum	Maximum	Mean value reduction	Median value reduction
T502-SIT-020	32	1.3100	0.83587	1.3500	0.63	1.62	0.02	3.35	42.10%	51.85%
T502-SIT-041	32	0.7584	0.58591	0.6500	0.26	1.13	0.00	2.51		
T502-SIT-045	32	0.9500	0.64446	0.8250	0.45	1.31	0.02	2.25	20.30%	21.21%
T502-SIT-059	32	0.7572	0.53315	0.6500	0.33	1.07	0.01	2.29		

Table S2. Descriptive statistics of the dSS for T502-SIT-020, T502-SIT-041, T502-SIT-045, and T502-SIT-059 (shorter regimen), showing mean/median reduction for the comparison between T502-SIT-020 and T502-SIT-041 or between T502-SIT-045 and T502-SIT-059.

Study	N	Mean	Standard deviation	Median	25th Percentile	75th Percentile	Minimum	Maximum	Mean value reduction	Median value reduction
T502-SIT-020	32	0.8363	0.46873	0.845	0.57	1.06	0.02	1.92	37.00%	43.79%
T502-SIT-041	32	0.5269	0.36890	0.475	0.25	0.70	0.00	1.28		
T502-SIT-045	32	0.5756	0.38756	0.550	0.27	0.78	0.02	1.68	1.47%	0.91%
T502-SIT-059	32	0.5672	0.36942	0.545	0.31	0.77	0.01	1.43		

Table S3. Descriptive statistics of the dMS for T502-SIT-020, T502-SIT-041, T502-SIT-045, and T502-SIT-059 (shorter regimen), showing mean/median reduction for the comparison between T502-SIT-020 and T502-SIT-041 or between T502-SIT-045 and T502-SIT-059.

Study	N	Mean	Standard deviation	Median	25th Percentile	75th Percentile	Minimum	Maximum	Mean value reduction	Median value reduction
T502-SIT-020	32	0.4744	0.49776	0.315	0.07	0.73	0	2.00	51.19%	71.43
T502-SIT-041	32	0.2316	0.39204	0.090	0.00	0.33	0	2.00		
T502-SIT-045	32	0.3747	0.42597	0.235	0.04	0.57	0	1.52	49.21%	74.47%
T502-SIT-059	32	0.1903	0.26486	0.060	0.00	0.29	0	1.00		

Table S4. Significance tests, with t-test for normally distributed data and Wilcoxon test for non-normally distributed data.

Transition from Placebo to Active Treatment	t-test	Wilcoxon test
dSS 020 - dSS 041	-	≤ 0.0001
dSS 045 - dSS 059	0.898	-
dMS 020 - dMS 041	-	≤ 0.001
dMS 059 - dMS 045	-	≤ 0.001
CSMS 020 - CSMS 041	≤ 0.001	-
CSMS 045 - CSMS 059	0.057	-

Table S5. Baseline characteristics.

		Cohort		
		**2020**	**2022**	**Total**
**Age**	Valid N	32	32	64
	Mean	37	38	37
	Standard Deviation	10	13	12
**Height**	Valid N	32	32	64
	Mean	172	173	172
	Standard Deviation	9	9	9
**Weight**	Valid N	32	32	64
	Mean	76.6	75.6	76.1
	Standard Deviation	17.7	15.1	16.4

Table S6. Demographic data (gender distribution and ethnic origin).

			Cohort		
			**2020**	**2022**	**Total**
**Gender**	Male	Count	17	13	30
		Column N %	53.1%	40.6%	46.9%
	Female	Count	15	19	34
		Column N %	46.9%	59.4%	53.1%
	Total	Count	32	32	64
		Column N %	100.0%	100.0%	100.0%
**Ethnicity**	Asian	Count	0	1	1
		Column N %	0.0%	3.1%	1.6%
	Caucasian	Count	32	31	63
		Column N %	100.0%	96.9%	98.4%
	Total	Count	32	32	64
		Column N %	100.0%	100.0%	100.0%
